# Circulating cell free HPVDNA in HPV positive head and neck cancer reveals fast responders and early peakers during treatment

**DOI:** 10.1038/s41598-025-33345-4

**Published:** 2025-12-19

**Authors:** Mark Zupancic, Madeleine Birgersson, Ourania N. Kostopoulou, Linda Marklund, Rusana Bark, Signe Friesland, Cecilia Jylhä, Emma Tham, Anders Näsman, Lars Sivars, Tina Dalianis

**Affiliations:** 1 Department of Oncology-Pathology, Karolinska Institutet, Stockholm, Sweden; 2 Medical Unit Head, Neck, Lung, and Skin Cancer, Theme Cancer, Karolinska University Hospital, Stockholm, Sweden; 3 Department of Surgical Sciences, Section of Otolaryngology and Head and Neck Surgery, Uppsala University, Uppsala, Sweden; 4 Division of Ear Nose and Throat Diseases, Department of Clinical Sciences Intervention and Technology, Karolinska Institutet, Stockholm, Sweden; 5 Department of Clinical Genetics, Karolinska University Hospital, Stockholm, Sweden; 6 The Department of Molecular Medicine and Surgery, Karolinska Institutet, Stockholm, Sweden; 7 Department of Clinical Pathology, Karolinska University Hospital, Stockholm, Sweden; 8 Department of Immunology, Genetics and Pathology, Uppsala University, Uppsala, Sweden; 9 Department of Oncology-Pathology, Karolinska Institutet, Bioclinicum J6:20, Karolinska University Hospital, 171 64 Stockholm, Sweden

**Keywords:** Tonsillar cancer, Base of tongue cancer, Oropharyngeal cancer, Head and neck cancer, Cancer of unknown primary, CUP, HPV, Cell-free HPV DNA, Digital droplet PCR, Biomarkers, Cancer, Oncology

## Abstract

The incidence of human papillomavirus-positive (HPV^+^) head and neck cancer (HNC), particularly oropharyngeal squamous cell carcinoma (OPSCC), is rising globally. Although radio- or chemo-radiotherapy yields better outcomes for patients with HPV^+^ tumors compared to those with HPV-negative tumors, these treatments do not cure all cases and are associated with significant side effects. This underscores the need for more personalized therapy. To help individualize treatment, we evaluated the presence of cell-free HPV DNA (cfHPV-DNA) in plasma collected before, during, and after therapy in patients with HPV^+^ HNC and correlated cfHPV-DNA levels with clinical characteristics, HPV genotype, and treatment response. 267 longitudinal plasma samples were collected from 83 patients with HPV^+^ HNC/cancer of unknown primary, at diagnosis/recurrence, during treatment and follow-ups, and tested for cfHPV-DNA with droplet digital PCR assaying for HPV16, 18, 33 or 35.39. 76/81 (93.8%) eligible diagnostic or recurrence samples tested positive for cfHPV-DNA, while a diagnostic sample was unavailable/or had inadequate cfDNA in 2/83 cases. cfHPV-DNA levels declined in most patients by 3–4 weeks post-radiotherapy initiation and became undetectable (fast responders) in approximately 30% of cases. By 3–6 months post-treatment, most patients had no detectable cfHPV-DNA. To date, no new recurrences have been documented, although two patients were unresponsive to therapy. Most HPV^+^ OPSCC patients were cfHPV-DNA-positive at diagnosis and cleared their cfHPV-DNA upon treatment, but the speed of clearance varied depending on tumor sub-site, patient characteristics and treatment. The data suggest that follow-up with cfHPV-DNA is a promising clinical approach and should be expanded to include more patients and longer time periods.

## Introduction

Human papillomavirus (HPV) is a well-established risk factor for certain head and neck cancers (HNC), particularly oropharyngeal squamous cell carcinoma (OPSCC), and contributes to the rising incidence of tonsillar and base of tongue squamous cell carcinomas (TSCC and BOTSCC, respectively)—the two predominant OPSCC subsites^1–13^. HPV-positive (HPV^+^) OPSCC is associated with significantly better outcomes compared to HPV-negative (HPV^–^) OPSCC after radiotherapy (RT) alone, or combined with surgery, or chemo-radiotherapy (CRT)^2,3,5,10,14–18^. However, despite these favorable outcomes, current treatments are associated with considerable toxicity and may be excessively intensive for certain patients while still failing to achieve a cure in others. This underscores the need for more individualized treatment approaches—introducing novel therapies for patients with a poor prognosis and pursuing treatment de-escalation when appropriate^16–18^. Accordingly, recent research efforts have focused on refining the classification of HPV^+^ status and identifying prognostic biomarkers, recurrent mutations, and driver genes, with the aim of enabling both the development of new therapies and the safe implementation of less aggressive regimens^18–34^.

In parallel, there is growing interest in using circulating cell-free HPV DNA (cfHPV-DNA) as a non-invasive marker to monitor treatment response and detect recurrence earlier. For example, droplet digital polymerase chain reaction (ddPCR)-based assays for cfHPV-DNA in plasma have shown promise in both HPV^+^ OPSCC and cervical cancer^35–40^. Despite these advances, such assays have notable limitations: not all patients are cfHPV-DNA-positive at diagnosis, and in some cases, cfHPV-DNA may be detected even in the absence of clinically confirmed recurrence^35–40^.

To contribute further evidence in this evolving field, we prospectively followed a cohort of patients with HPV^+^ HNC - predominantly HPV^+^ OPSCC - from 2024 onward in the Stockholm and Gotland Regions. Plasma samples were collected at diagnosis, during treatment, and throughout regular follow-up, and cfHPV-DNA levels were measured using a validated ddPCR method^37,40^. Importantly, we correlated cfHPV-DNA levels with key clinical and tumor characteristics, including age, smoking history, treatment modality, tumor subtype, and tumor HPV type. We believe these correlations enhance the interpretation of our findings and highlight both the benefits and limitations of implementing cfHPV-DNA testing in clinical practice.

## Materials and methods

### Cohort

Patients diagnosed with HPV^+^ HNC (International Classification of Diseases 10th Revision (ICD-10): TSCC: C09.0-9 and C02.4; BOTSCC: C01.9; cancer of unknown primary (CUP): C80.0, malignant tumor of the lacrimal duct C69.5 and nasopharyngeal carcinoma: C11.3 in the Stockholm and Gotland Region, treated at the Karolinska University Hospital in Stockholm, were included in the study. More specifically, from February 2024 all HPV^+^ HNC patients diagnosed at the Karolinska University Hospital, Stockholm, Sweden, were informed about this prospective study, approved by the Swedish Ethical Review Authority (permission 2023-04595-01). Informed written consent was obtained from all patients included in the study. So far, we have analyzed 267 samples from 83 patients who have agreed to participate in this ongoing study.

The data below include an interim presentation of the study so far. All methods were carried out in accordance with relevant guidelines and regulations.

In primary tumors, HPV^+^ status was defined as the tumor being HPV-DNA positive by a PCR assay and overexpressing p16INK4a (p16^+^) in > 70% of the cells by immunohistochemistry and these data were obtained from patient case reports^41–43^. In patients with a cancer of unknown primary of the head and neck region (HNCUP), presence of HPV DNA was sufficient, since p16 analysis was not always possible to perform. The patients and some of their tumor characteristics are presented in Table 1.

**Table 1:**
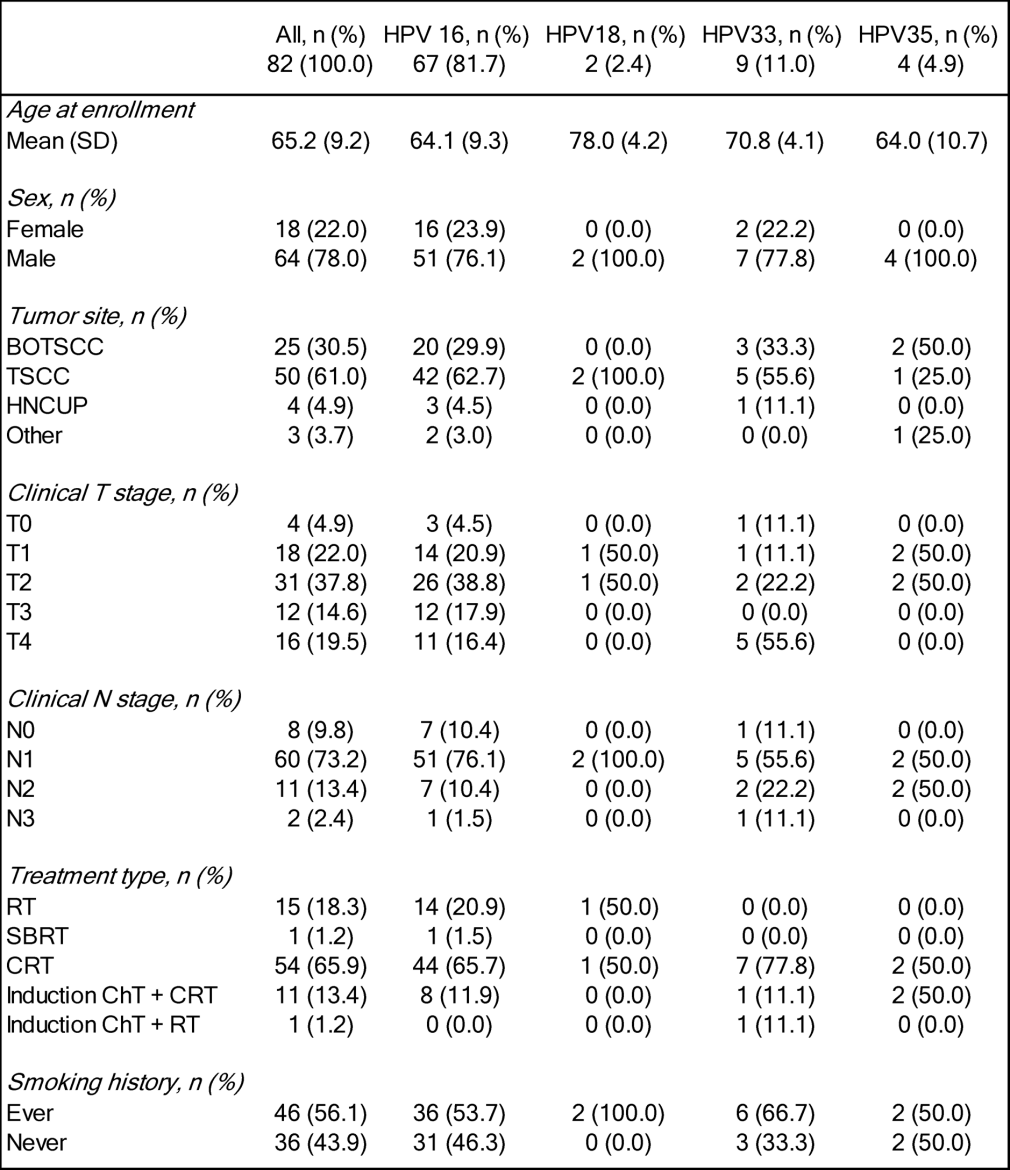
Characteristics of patients with human papillomavirus-positive head and neck cancer included in the study. BOTSCC:base of tongue squamous cell carcinoma; TSCC:tonsillar squamous cell carcinoma; HNCUP:cancer of unknown primary of the head and neck region; RT:radiotherapy; SBRT:stereotactic body radiotherapy; CRT:chemoradiotherapy; ChT:chemotherapy.

### Plasma samples: collection and storage

Blood samples were collected at diagnosis, 3–4 weeks after RT initiation, and subsequently at 3, 6, 9, and 12 months after treatment completion. On each occasion, two blood samples were collected using Cell-Free DNA (cfDNA) BCT tubes (STRECK, La Vista, NE, USA), which preserve circulating cfDNA for up to 14 days at temperatures ranging from 6 °C to 37 °C. The tubes were handled within 8 days of collection, at Bioclinicum, a joint research laboratory facility of the Karolinska University Hospital and Karolinska Institutet. The tubes were centrifuged 1,600 × g for 10 min at 4 °C. The supernatant was then transferred to 5 mL Eppendorf DNA LoBind tubes (Merck Life Science UK, Limited, Dorset, UK) and further centrifuged at 3200 × g for 10 min at 4 °C. The resulting supernatant was collected and stored at -80 °C in plastic cryovials (SARSTEDT AG & Co. KG, Nümbrecht, Germany) until further processing.

### Therapy options

Most patients (~ 80%) were treated with CRT or induction chemotherapy (ChT) and RT (Table 1), while RT alone was only given to a minority of the patients. RT was administered as intensity-modulated radiation therapy, delivered in 2 Gray (Gy) fractions to a total dose of up to 68 Gy, 5–6 fractions per week. When ChT was administered as part of CRT, the agents used included cisplatin (40 mg/m^2^), carboplatin (AUC2, calculated using the Calvert formula), docetaxel (15 mg/m^2^), or cetuximab (loading dose: 400 mg/m², followed by 250 mg/m²). The agents were given weekly (Q1W). For induction therapy, the following regimens were used: carboplatin (AUC6, Calvert formula) plus paclitaxel (200 mg/m²), cisplatin (80 mg/m²) plus gemcitabine (1000 mg/m²), and cisplatin (75 mg/m²) plus docetaxel (75 mg/m²). Induction ChT was administered every 3 weeks (Q3W). In patients with > N0 disease a Positron-Emission-Tomography scan and Computed Tomography (PET-CT) was done 3–4 months after completed therapy.

###  cfDNA extraction

In most cases, cfDNA was isolated from 3 to 4 mL of plasma per sample. However, in 25 out of 267 samples (9.4%), less than 3 mL of plasma was available. Extraction was performed using the QIAamp Circulating Nucleic Acid Kit (Qiagen, Hilden, Germany), following the manufacturer’s instructions. DNA was eluted in 40 µL of AVE buffer, as previously described^37,40^.

###  Digital droplet PCR

cfDNA samples were analyzed using ddPCR on the QX200 ddPCR system, following the manufacturer’s instructions (Bio-Rad, Hercules, CA, USA), as previously described^37,40^. For each patient, only the HPV type found in the corresponding tumor biopsy was analyzed, using ddPCR assays targeting the *E7* gene of HPV16, HPV18, HPV33, and HPV35^37,40^. A separate ddPCR assay targeting cell-free human albumin DNA (cfALB) was used as a control for total cfDNA in the blood^37,40^. For HPV16 and HPV18, cfDNA derived from cervical cancer patients was used as a positive control, while plasmid constructs served as controls for HPV33 and HPV35^37,40^. All samples were run in triplicates using 11 µL of eluted cfDNA, 5 µL of ddPCR Multiplex Supermix, 900 nmol/L primers, and 250 nmol/L probes per well. Reactions were brought to a final volume of 20 µL with nuclease-free water. Droplet counts from all three wells were pooled, and Poisson statistics were applied to calculate the concentration and quantify cfHPV-DNA. The average of two replicates was used (*n* = 2) if one of the triplicates failed; however, if only one replicate remained, the sample was rerun. For additional methodological details, see references^37,40^. Normal control samples were obtained from anonymized blood donors and processed as described above, using peripheral blood plasma extracted with the QIAamp Circulating Nucleic Acid Kit (Qiagen, Hilden, Germany). Non-template controls containing nuclease-free water served as blanks^37,40^. A sample was classified as positive for cfHPV-DNA (HPV16, HPV18, HPV33, or HPV35) if ≥ 3 droplets were positive for the respective HPV type. Samples with cfALB levels below 333 copies/mL were excluded from analysis^37,40^.

### Statistical analysis

All statistical analyses were performed using IBM SPSS Statistics software (version 29.0; Armonk, NY, USA). Categorical variables were compared using the chi-squared test, while continuous variables were assessed using the independent samples t-test. A two-tailed, unpaired Student’s t-test was used to compare mean age and median HPV copy number between groups. To evaluate differences in cfHPV-DNA levels, non-parametric tests—including the Mann–Whitney U or Kruskal–Wallis test—were applied, as appropriate. Chi-squared tests were also employed to compare early and late treatment responders. All p-values were two-sided, and statistical significance was defined as *p* < 0.05.

## Results

### Patient cohort, patient and tumor characteristics, and HPV type

#### Patients and samples

**Fig. 1:**
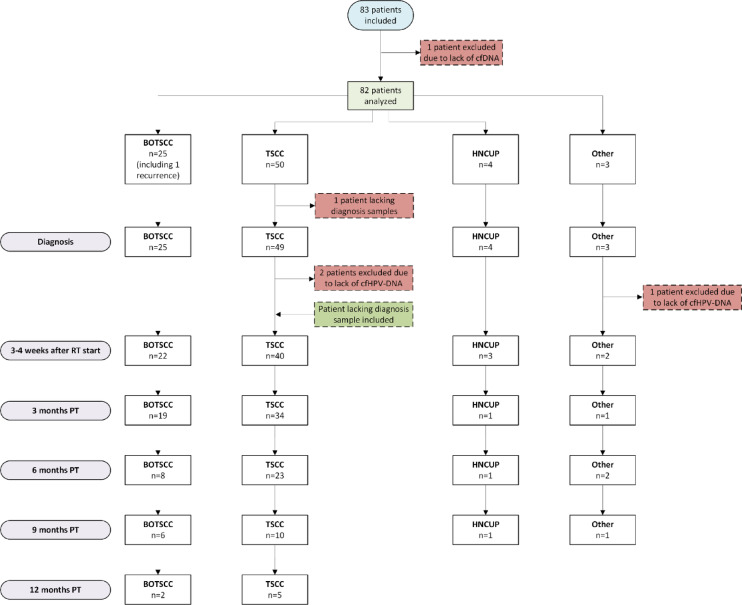
Flow chart of patient inclusion and exclusion. Total number of patients and number of included patients per tumor site, including the number of patients per site and time point. Of note, an addition to the indicated exclusions and inclusions, not all patients have samples at each time point after the diagnosis sample. BOTSCC: base of tongue squamous cell carcinoma; TSCC: tonsillar squamous cell carcinoma; HNCUP: cancer of unknown primary of the head and neck region; PT: post completed therapy.

Samples from 83 patients with HPV^+^ HNC or HNCUP were collected over a 15-month observation period as part of an ongoing study. As a result, samples were not available at all time points for every patient. One patient with TSCC was excluded due to failed cfDNA extraction, as shown in the study flowchart (Fig. 1). To date, no explanation has been identified for this extraction failure. Among the remaining 82 patients analyzed, 78 had HPV^+^ HNC - including one case of recurrent BOTSCC - and four had HPV^+^ HNCUP (Table 1; Fig. 1).

####  Tumor subtypes

Among the 82 patients included, 50 (61.0%) had TSCC, 25 (30.5%) had BOTSCC (including one patient with recurrence), four (4.9%) had HNCUP, and three (3.7%) had other cancers, including one carcinoma of the lacrimal duct and two nasopharyngeal carcinomas (Table 1; Fig. 1). All primary tumors were positive for HPV DNA and overexpressed p16^ink4a^ (p16^+^), including the recurrent BOTSCC tumor^41–43^. All four HNCUP cases were HPV-DNA-positive; however, p16 immunochemistry was not routinely performed in this subgroup (Table 1 and data not shown).

#### Patient’s age and smoking habits

At diagnosis, the mean patient age was 65 years. Most patients had HPV16^+^ tumors, with a mean age of 64 years. In contrast, patients with HPV33^+^ tumors were significantly older, with a mean age of 71 years (*p* = 0.039). A similar trend was observed in the two patients with HPV18^+^ tumors, who had a mean age of 78 years. Patients with HPV35^+^ cancer did not differ significantly in age from those with HPV16^+^ tumors. Overall, 43.9% of patients were never-smokers, while 56.1% were ever-smokers, with a median age of 63 and 67 years, respectively (*p* = 0.123).

#### Presence of cfHPV-DNA at diagnosis, in correlation with patient characteristics, tumor subtype, tumor size, nodal stage, HPV type, and smoking

##### Tumor subtypes and presence of cfHPV-DNA at diagnosis

cfHPV-DNA was detected in 76 of 81 (93.8%) diagnostic plasma samples (Table 2). One of the 82 patients included in the study who lacked a diagnostic sample, contributed post-diagnostic samples and was, therefore, retained in subsequent analyses (Fig. 1). Five patients were cfHPV-DNA-negative at diagnosis. Specifically, cfHPV-DNA was detected at diagnosis in 47 of 49 (95.9%) patients with TSCC (one diagnostic sample was unavailable), 24 of 25 (96.0%) with BOTSCC (including one recurrence), all 4 of 4 (100%) with HNCUP, and 1 of 3 (33.3%) with other non-TSCC/BOTSCC HNCs. At diagnosis, the median cfHPV-DNA level was 206.3 copies/ml. When stratified by tumor type, the median values were 758.9 copies/mL for HNCUP, 322.1 copies/mL for BOTSCC, and 183.9 copies/mL for TSCC (*p* = 0.603).

**Table 2:**
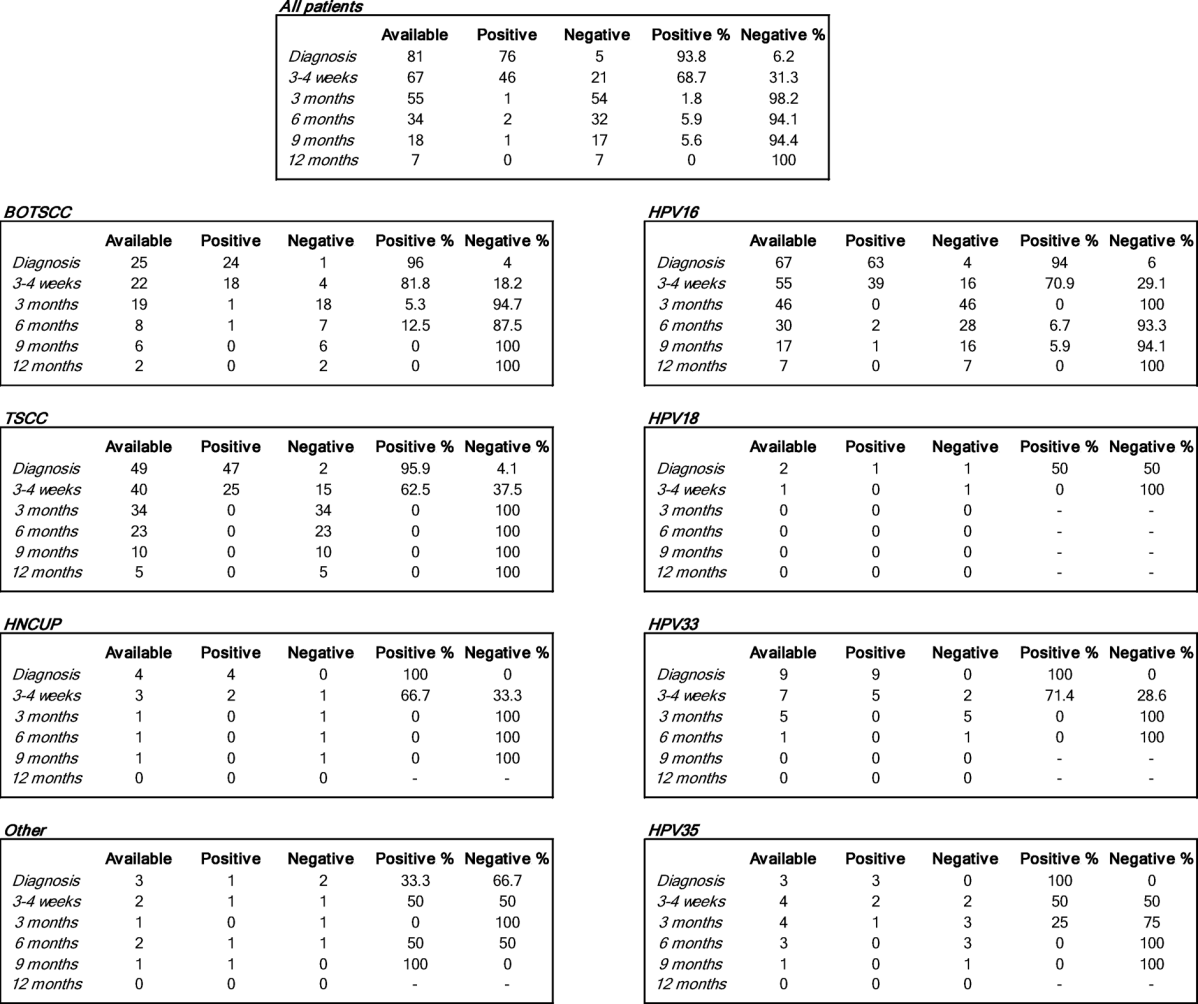
Available patient samples and the number of cfHPV-DNA positive and negative samples at each sampling time point per HPV type and tumor site. BOTSCC: base of tongue squamous cell carcinoma; TSCC: tonsillar squamous cell carcinoma; HNCUP: cancer of unknown primary of the head and neck region.

##### Tumor and nodal stage, smoking and presence of cfHPV-DNA at diagnosis

Tumor and nodal stage and history of smoking for all patients is presented in Table 1. There was a tendency for tumors with T1-stage to have less cfHPV-DNA at diagnosis than higher T-stages (T1: 37.6, T2: 229.7, T3: 228.7 and T4: 316.0 median copies/ml, respectively). However, this difference did not reach statistical significance when comparing T1 with all other T-stages (*p* = 0.126, Mann-Whitney U-test).

Further analysis was conducted for the five patients who were cfHPV-DNA-negative at diagnosis. Among them, four were HPV16^+,^ and one was HPV18^+^. The T-stage were as follows: the HPV18^+^ TSCC and one HPV16^+^ nasopharyngeal carcinoma were both T1; HPV16^+^ TSCC and BOTSCC were T2 and T3, respectively; and the HPV16^+^ lacrimal duct carcinoma was T4b. These findings did not reveal a clear correlation between tumor stage and absence of cfHPV-DNA at diagnosis.

Interestingly, 4/5 (80%) patients with cfHPV-DNA negative plasma at diagnosis had N0-disease. There was also a tendency for N0-stage disease to correlate to a lower cfHPV-DNA level (N0: 26.3, N1: 217.8, N2: 253.2, N3: 703.0 median copies/ml, respectively), but this failed to reach significance (*p* = 0.111) when comparing N0 to all other N-stages. Notably, taking all HPV16^+^ OPSCC into account, none with ≥N1 were cfHPV-DNA negative at diagnosis.

Presence of cfHPV-DNA at diagnosis could not be correlated to smoking status (*p* = 0.88).

#### Presence of cfHPV-DNA at diagnosis and during and after treatment, in correlation to patient characteristics, tumor subtype, tumor and nodal stage, and HPV type

##### HPV types and presence of cfHPV-DNA

Data have been included for 82 patients, but since this is an interim study, samples were not available for all patients at all time points (Fig. 1). Table 2 gives an overview of available patient samples and the number of cfHPV-DNA-positive and negative samples at each time point. Most HNC/HNCUP were HPV16^+^ (67/82, 81.7%). The remaining patients had HNC/HNCUP that were positive for HPV18 (2/82, 2.4%), HPV33 (9/82, 11%), and HPV35 (4/82, 4.9%) (Table 1). The result for each HPV type is described below.

##### Patients with HPV16^+^ cancer

In total 67 patients in the cohort had HPV16^+^ cancer, and of these, 42/67 (62.7%) were TSCC, 20/67 (29.9%) were BOTSCC (including the recurrence), 3/67 (4.5%) were HNCUP and 2/67 (3%) had tumors of other primaries. Among these patients, 63/67 (94%), were cfHPV-DNA-positive in their diagnostic sample, and two more patients became cfHPV-DNA-positive at three weeks after initiation of RT, i.e. 65 patients were cfHPV-DNA positive at an early time point (at diagnosis and during treatment); and of these, 58 patients had follow-up samples (Table 2).

**Fig. 2:**
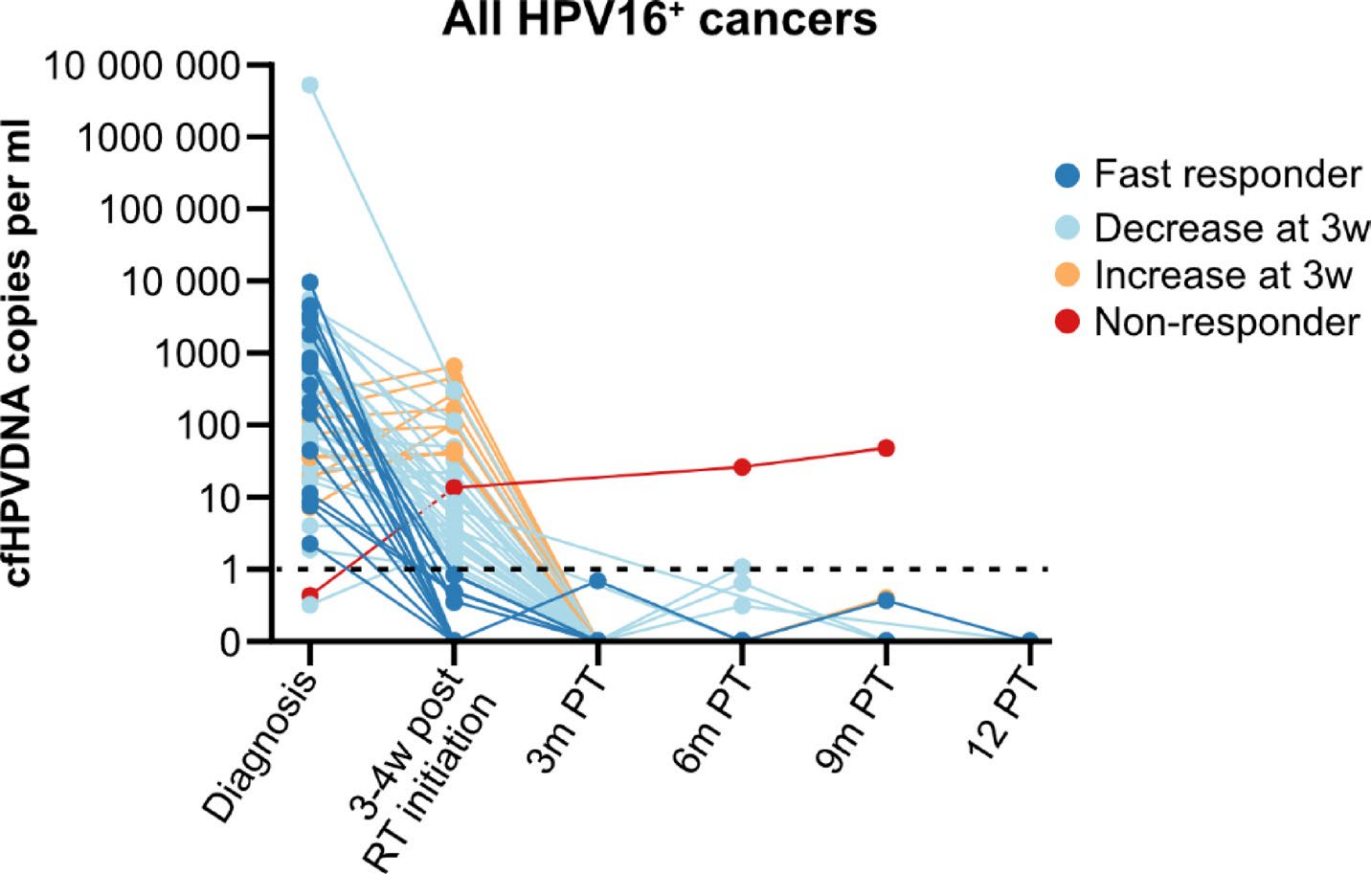
cfHPV-DNA levels in patients with HPV16^+^ head and neck cancer/cancer of unknown primary of the head and neck region up to 12 months after completed treatment. Response in all 58 patients with positive cfHPV16-DNA at screening or 3–4 weeks after RT initiation. The data are presented as HPV16 copies per ml, where levels below 1 copy per ml are considered negative, as indicated by the dotted line. The colors of the lines represent the response to treatment of each of patient. RT: Radiotherapy; W: weeks; PT: post completed therapy.

**Fig. 3:**
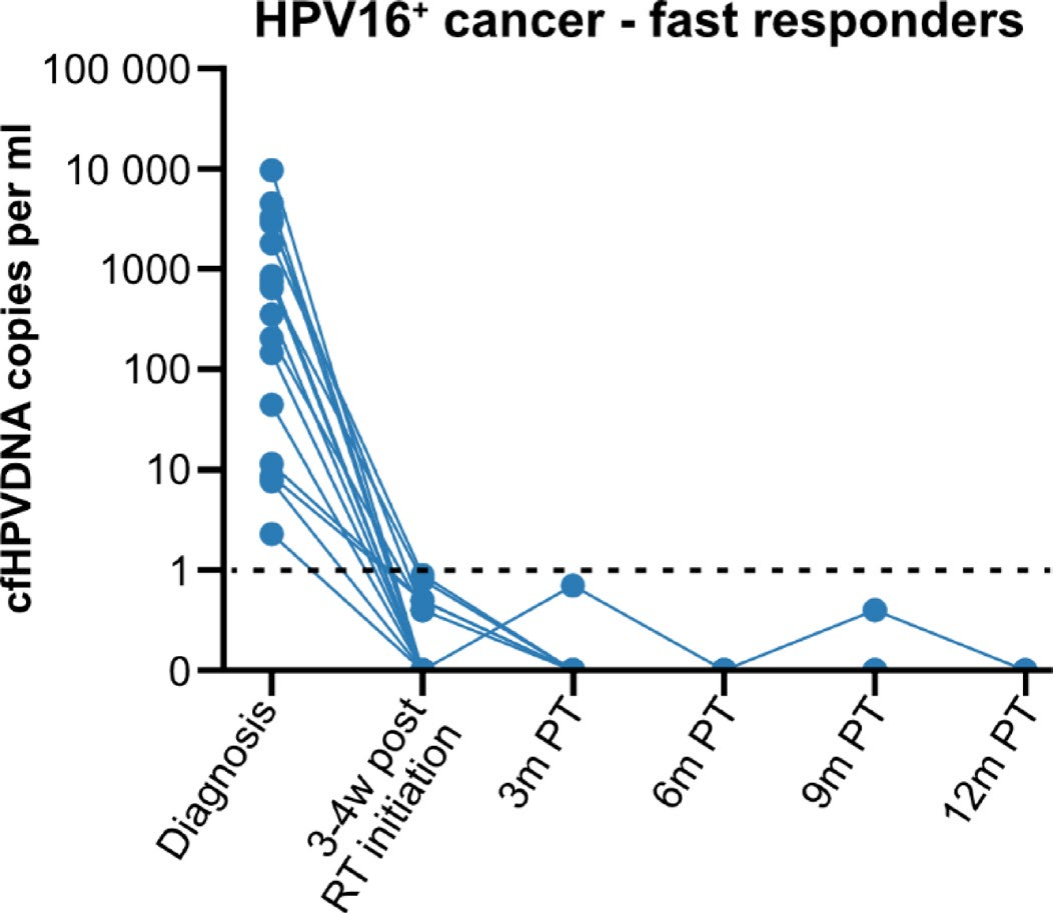
cfHPV-DNA levels in patients with HPV16^+^ head and neck cancer/cancer of unknown primary of the head and neck region with a fast response after treatment. cfHPV16-DNA levels in the 15 patients who had non-detectable levels already halfway through their radiotherapy treatment. The data are presented as HPV16 copies per ml, where levels below 1 copy per ml are considered negative, as indicated by the dotted line. RT: Radiotherapy, W: weeks; PT: post completed therapy.

**Fig. 4:**
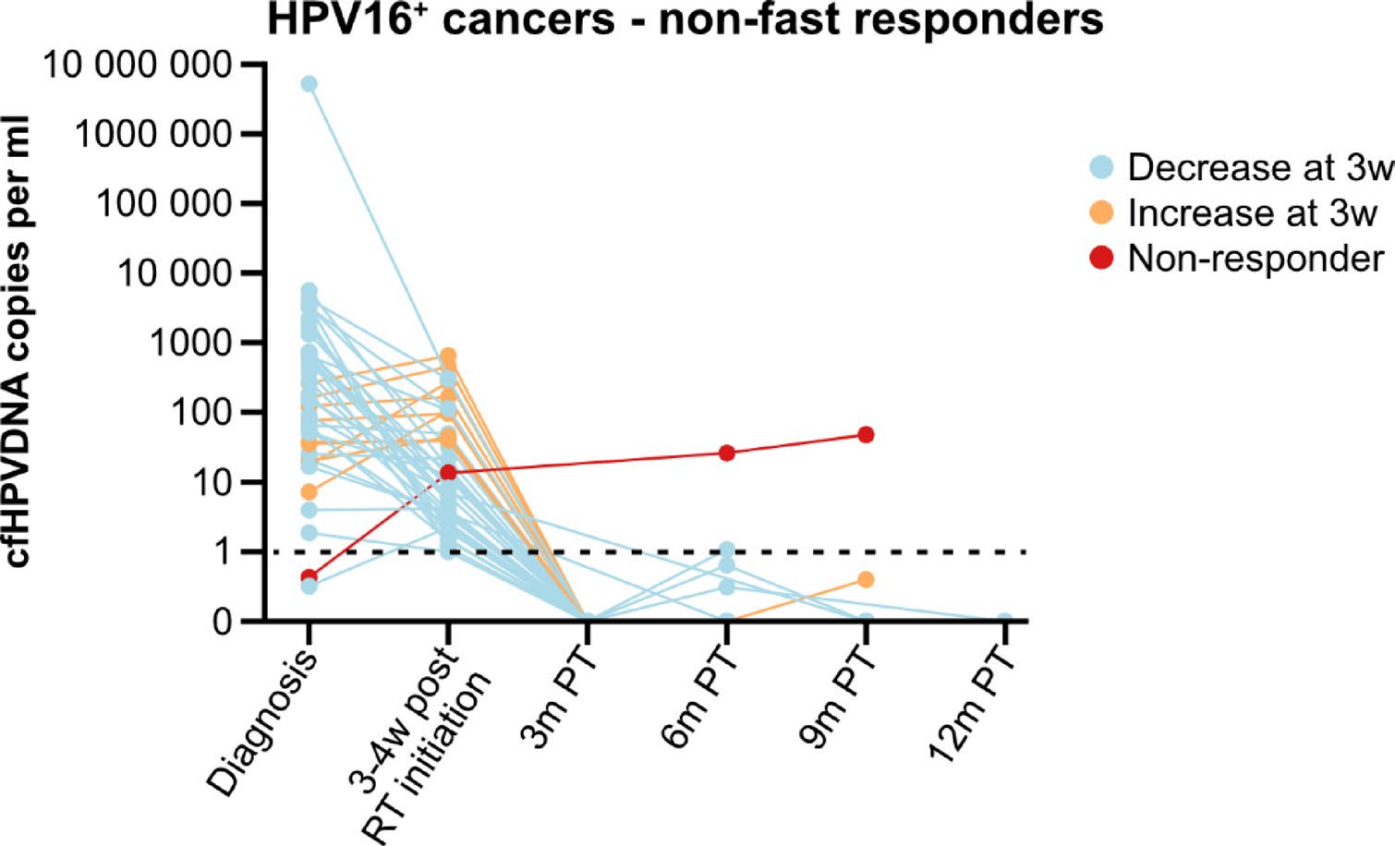
cfHPV-DNA levels in patients with HPV16^+^ head and neck cancer/cancer of unknown primary of the head and neck region with a slower or no response to treatment. cfHPV16-DNA levels in the 43 patients who still had detectable levels 3–4 weeks after start of radiotherapy. The data are presented as HPV16 copies per ml, where levels below 1 copy per ml are considered negative, as indicated by the dotted line. The colors of the lines represent the response to treatment of each patient. RT: Radiotherapy; W: weeks; PT: post completed therapy.

Responses to treatment are depicted in Figs. 2, 3 and 4, which show cfHPV-DNA levels over time, and include only the 58 HPV16^+^ cancer patients who were cfHPV-DNA-positive at diagnosis or 3 weeks post-RT initiation. More specifically, Fig. 2 includes all 58 patients, while Fig. 3 includes 16 patients with a fast cfHPV-DNA decline i.e. (fast responders), with no cfHPV-DNA already 3 weeks after initiation of RT, while Fig. 4 depicts all remaining patients (non-fast responders).

Data at 3 weeks after initiated RT were available for 55 patients, and at this time point 16/55 (29.1%) patients had no measurable cfHPV-DNA in plasma already halfway through the RT treatment (fast responders) (Fig. 3). Moreover, at this time point, a decrease in cfHPV-DNA levels was observed for most, but not all, of the remaining patients (Figs. 2, 3 and 4; Table 2).

Data at 3 months after completed therapy were available for 46 patients, and at this time point 46/46 (100%) of the patients had no measurable cfHPV-DNA (Figs. 2, 3 and 4; Table 2). Data 6, 9 and 12 months, respectively, after completed therapy data were available for 30, 17 and 7 patients, respectively (Table 2). Most patients with available samples 6 and 9 months after treatment were cfHPV-DNA-negative, with one exception. As mentioned earlier, the patient with the HPV16^+^ lacrimal duct cancer had a very low value (i.e., below the pre-set cut-off) of cfHPV-DNA in plasma at diagnosis. The cfHPVDNA values have since then risen both during, as well as at 6 and 9 months after completed treatment, but so far, no clinical recurrence has been diagnosed. However, no sample was available at 3 months post completed treatment for this patient. For further details, see the group reports.

**Fig. 5:**
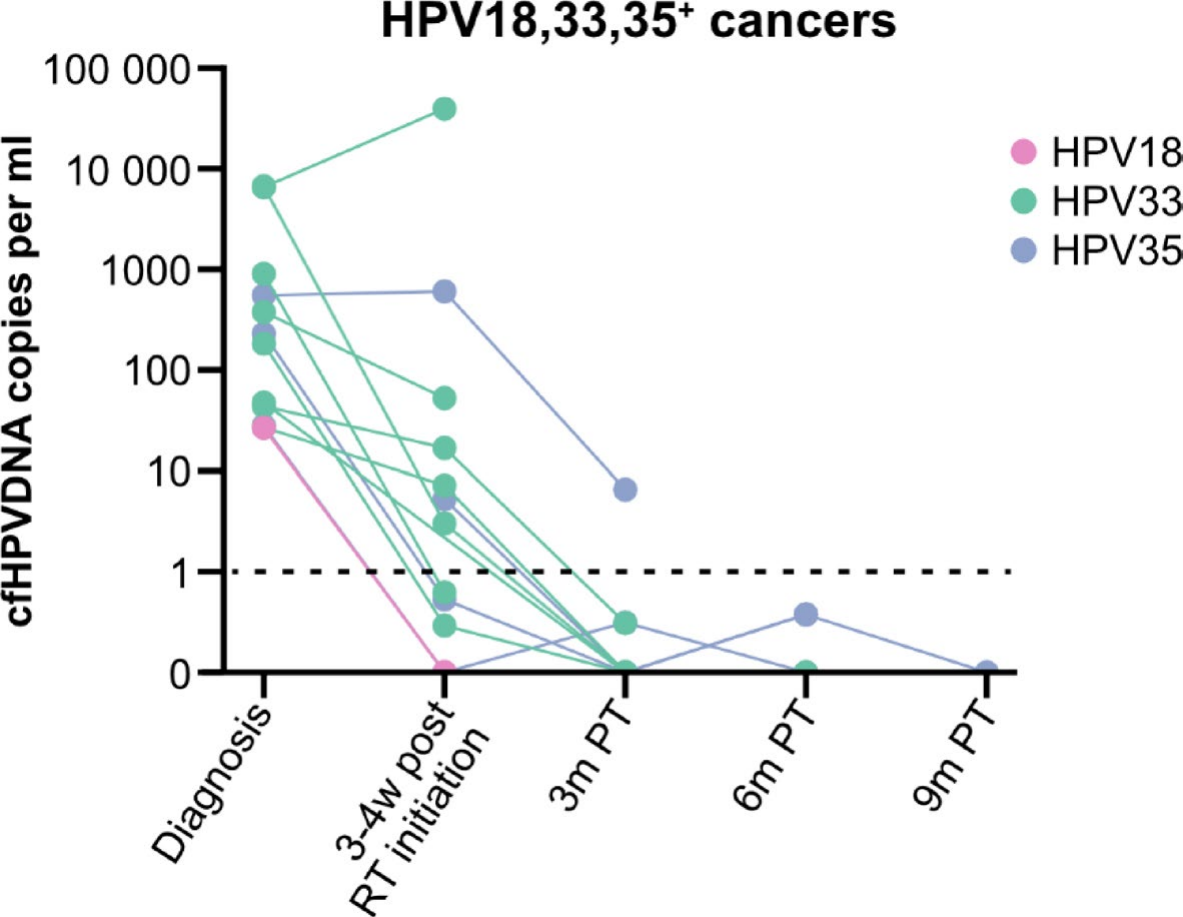
cfHPV-DNA levels in patients with HPV18, 33, or 35^+^ head and neck cancer/cancer of unknown primary of the head and neck region up to 9 months after completed treatment. Response in 13 patients with positive cfHPV-DNA at screening or 3–4 weeks after RT initiation (HPV18 = 1, HPV33 = 8, HPV35 = 4). The data are presented as HPV18, 22 or 35 copies per ml, where levels below 1 copy per ml are considered negative, as indicated by the dotted line. The colors of the lines represent the HPV type. RT: Radiotherapy; W: weeks; PT: post completed therapy.

*Patients with HPV18*^*+*^, *HPV33*^*+*^, *and HPV35*^*+*^ *cancers.* The response patterns among patients with HPV18^+^, HPV33^+^, and HPV35^+^ cancers were analogous to those observed in HPV16^**+**^ patients (Fig. 5).

At diagnosis, 15 patients with HPV18^*+*^, HPV33^*+*^, and HPV35^+^ cancers had available samples, and 14 (93.3%) of them were cfHPV-DNA-positive (Table 2). The exception was a patient with HPV18^+^ TSCC with early-stage disease (T1N0M0).

At 3 weeks post-RT initiation, samples were available for 12 patients; of these, five (41.7%) were cfHPV-DNA-negative. All but two patients showed a decrease in cfHPV-DNA levels. The exceptions were an HPV35^**+**^ BOTSCC patient and an HPV33^**+**^ TSCC patient, both of whom exhibited progressive disease and resistance to treatment (Fig. 5; Table 2).

At 3 months post-treatment, samples from nine patients were available, and eight of them (88.9%) were cfHPV-DNA-negative. The remaining patient, who had HPV35^**+**^ cancer and an inadequate response to treatment, showed a slow decline in cfHPV-DNA levels (Fig. 5). At 6 and 9 months after completed therapy, samples were available from four and one patient, respectively, and these were all cfHPV-DNA negative (Fig. 5; Table 2). However, to date, the patient above with the HPV35^**+**^ cancer has not donated a later sample.

### Fast responders, late responders and patients disclosing an early cfHPV-DNA peak defined as “early peakers”

Patients with TSCC were more likely to be early responders to treatment, i.e., with cfHPV-DNA cleared at 3–4 weeks, compared to patients with BOTSCC. More specifically, 15/40 (37.5%) of TSCC had cleared cfHPV DNA at 3–4 weeks compared to only 3/21 (14.3%, excluding the one recurrence) of BOTSCC patients (Table 2, *p* = 0.059). Furthermore, fast TSCC/BOTSCC responders presented with T1 disease more frequently (8/18, 44.4%) than late responders (5/43, 11.6%, *p* = 0.004).

There were no significant differences between fast- and non-fast responders with regard to cfHPV-DNA levels at diagnosis, or with regard to age or N-stage both (*p* = 0.519, *p* = 0.682 and *p* = 0.160 respectively). Furthermore, although roughly one third of TSCC cases had undergone tonsillectomy before treatment, there was no difference in the frequency of tonsillectomy among TSCC fast-resonders as compared to other TSCC patients.

A swifter cfHPV-DNA decline did not appear to be correlated entirely to treatment and was observed in patients given CRT with/without induction ChT (data not shown). A swift cfHPV-DNA decline was also observed in one patient given RT alone. However, looking only at patients who received CRT with/without induction ChT, patients given induction are more likely to be fast responders (*p* = 0.0428).

Non-fast responders could be further subdivided into “early peakers” (i.e., those with an initial increase in cfHPV-DNA at 3–4 weeks post-RT initiation) and those with a decrease in cfHPV-DNA that did not result in complete clearance by that time. “Early peakers” were significantly older than other non-fast responders (mean age 72.05 and 61.71 years; *p* < 0.001) and had significantly lower cfHPV-DNA levels at diagnosis (36.8 vs. 609.3 copies/ml, *p* < 0.001). When comparing “early peakers” with all other patients (including early responders), “early peakers” were still significantly older (72.05 vs. 64.04, *p* = 0.003) and had lower cfHPV DNA levels at diagnosis (median 36.8 vs. 427.9 copies/ml, *p* = 0.008). No differences were observed for TSCC vs. BOTSCC, T or N stage, or HPV types when comparing “early peakers”, either with other non-fast responders or with all other patients (data not shown).

### Group reports

When considering only patients with HPV^+^ and p16-positive HNC (excluding HNCUP cases), several notable group-level observations emerged.

*Of the three non-OPSCC patients*, only one was cfHPV-DNA positive at diagnosis. This individual had HPV35^**+**^ nasopharyngeal carcinoma (T1N2M0) with a baseline cfHPV-DNA value of 28 copies/ml, which subsequently cleared rapidly after treatment. A second patient with HPV16^**+**^ lacrimal duct carcinoma (T4bN0M0) was initially cfHPV-DNA negative (0.4 copies/ml, below the pre-set cut-off for positivity) but demonstrated positive values at 6 and 9 months post-treatment (26.6 and 48.1 copies/ml, respectively). Despite this rise, clinical relapse has not yet been confirmed, although continued close follow-up is warranted. The third patient, with HPV16^**+**^ nasopharyngeal carcinoma (T1N0M0), remained cfHPV-DNA-negative throughout the observation period and showed a favorable response to treatment.

In summary, the utility of cfHPV-DNA as a marker of minimal residual disease in patients with non-OPSCC HNC requires further evaluation.

#### Among the OPSCC patients, two were organ transplant recipients

Neither of these patients had exceptionally high cfHPV-DNA levels at diagnosis; however, both experienced poor clinical outcomes, including significant difficulty tolerating treatment. One patient, diagnosed with HPV16^+^ BOTSCC, failed to respond to therapy and demonstrated rising cfHPV-DNA levels during and after treatment. This patient died shortly after completing therapy, presenting with progressive disease. The other patient, diagnosed with HPV33^+^ TSCC, provided only the diagnostic sample and died shortly after completing treatment, although without evident disease progression.

In summary, organ transplant recipients may not present with markedly high cfHPV-DNA levels at diagnosis, but in this study they appeared particularly vulnerable to the intensity of treatment for OPSCC and may not tolerate it well. Nevertheless this too requires further evaluation.

#### Reflections on patients with TSCC/BOTSCC with unexpected cfHPV-DNA response

 Below responses of four TSCC/BOTSCC patients are highlighted.

One BOTSCC patient who presented with an en passant lesion in the hip 3 years after completing therapy was later diagnosed with a distant recurrence. This patient had a high cfHPV-DNA level at baseline, supporting the interpretation that the lesion represented an HPV^+^ bone metastasis. The metastasis was confirmed through HPV analysis of a tissue biopsy (data extracted from patient records). Upon treatment, regression was observed, and the cfHPV-DNA levels dropped to zero.

Another patient, with an HPV16^+^ TSCC, was cfHPV-DNA positive upon diagnosis, responded swiftly and was cfHPV-DNA-negative 3 weeks post initiation of RT, and had a cfHPV-DNA level of 0.7 copies/ml (below the positive threshold) 3 months after completed therapy. Still, a PET-CT scan performed 3 months after completed treatment showed remaining metabolic activity in the mass on the neck. The patient underwent a neck dissection, and the pathology report confirmed a single lymph node with viable cancer cells. This suggests that some caution is needed and a possible reconsideration as to whether the positive threshold is adequate, or e.g. additional samples should be tested when finding a near-threshold cfHPV-DNA positive value.

Two additional patients, one with an advanced HPV35^+^ BOTSCC and the other with an advanced HPV16^+^ TSCC, both with high cfHPV-DNA levels at diagnosis were also monitored. Their cfHPV-DNA levels decreased upon treatment, and correlated with treatment response, especially in the patient with the HPV16^+^ TSCC, now in remission with undetectable cfHPV-DNA levels for several months after completed treatment. The patient with an HPV35^+^ BOTSCC also presented an initial decrease, but cfHPV-DNA was still detectable at 3 months after therapy and the disease has progressed. This patient is now on palliative treatment, but no further samples have been analyzed so far.

In summary, this final group of patients illustrates the heterogenous responses to treatment, and demonstrates that a decrease in cfHPV-DNA levels generally aligns with clinical improvement, but it also underscores that caution is needed in interpreting the data of samples with detectable cfHPV-DNA just below the threshold for positivity.

## Discussion

In this study, 82 HPV^+^ HNC/HNCUP patients, with 267 longitudinal plasma samples, were followed by ddPCR to monitor cfHPV-DNA at diagnosis, during treatment and at regular follow-ups in order to assess its clinical reliability for monitoring disease regression or progression.

cfHPV-DNA assayed by ddPCR was detected at diagnosis in 76/81 (93.8%) of HNC/HNCUP cases, including the one recurrence. Moreover, already 3 weeks after RT initiation, a complete decrease in cfHPV-DNA levels was observed in almost one third of the patients (21/67, 31.3%), and by 3 months post completed treatment, this was true for almost all tested patients (54/55, 98.2%) (Table 2). Nonetheless, during the 6–12 month observation period, two patients did not reach negative cfHPV-DNA levels, and these are discussed in further detail below.

The decrease of cfHPV-DNA was also followed over time in correlation to initial cfHPV-DNA levels in plasma at diagnosis, tumor subtype, tumor stage, patient age, treatment and HPV type. In this study, cfHPV-DNA levels tended to correlate to N-stage and T-stage, as well as tumor subtype (HNCUP > BOTSCC > TSCC), however none of these associations reached statistical significance. Nevertheless, the observed trend toward higher cfHPV-DNA levels with increasing T and N stages, with emphasis on nodal stage, is consistent with prior findings in cervical and oropharyngeal cancers, as well as with results from recent systematic reviews^37,38,40,44–48^.

In addition, it was possible to identify patients with both HPV16 and non-HPV16 genotypes as either fast or non-fast responders. The fast responders exhibited undetectable cfHPV-DNA levels at 3–4 weeks of initiating RT, whereas in the remaining patients, cfHPV-DNA clearance occurred later. Comparing these two groups, we observed that the early disappearance of cfHPV-DNA levels did not necessarily correlate with an initially low cfHPV-DNA in the diagnostic sample, a small tumor size, or treatment. Moreover, although some patients had obtained induction ChT prior to RT (i.e. CRT or RT), this did not apply to all. Nevertheless, we did note that patients with TSCC tended to respond faster than those with BOTSCC (irrespective of the former having undergone a diagnostic tonsillectomy or not), as did patients with T1 disease. To our knowledge, there are limited reports on the distinction between fast and non-fast responders in HPV^+^ OPSCC^46,47^. Whether a fast response pattern is attributable to specific subgroups of patients e.g. who are younger and have a more efficient immune system, or qualities of the tumor or specific treatments, remains to be investigated further. However, we anticipate similar to the conclusions of Chera et al.^46^, that fast responders could be a cohort where treatment de-escalation could be proposed, especially if other favorable biomarkers are disclosed in parallel^49,50^.

Of note, we also identified a subgroup of patients with an initial increase in cfHPV-DNA here referred to as “early peakers”. This phenomenon has been reported previously in cervical cancer patients as well as in HPV^+^ OPSCC patients^40,46,47^. These “early peakers” were in this study significantly older than both the early and typical responders, and had lower cfHPV-DNA levels at diagnosis. The underlying reason for this type of response is not clear, but it is possible that older patients have weaker immune systems, which could impact the clearance of the tumor and cfHPV-DNA levels. The recognition of this “early peaker” subgroup could be clinically relevant, as an initial increase in cfHPV-DNA in older patients should not necessarily be interpreted as treatment failure. It should however be noted, that in the study by Chera et al., among patients defined as having “an unfavorable clearance profile” so called “early peakers” could be included, and there those with adverse clinical markers often experienced a high rate of residual or recurrent regional disease^46^. This was nevertheless, not the case in the study by Cao et al., where instead a low initial cfHPV-DNA combined with an early increase was a positive factor^47^. Clearly, further investigations are required to explore the clinical impacts associated to the observations of “early peakers” and “unfavorable responders.”

cfHPV-DNA analysis was done separately for patients with HPV16^+,^ 18^+^, 33^+^ and 35^+^ cancer. High detection frequencies were found in the respective diagnostic samples of patients with HPV16^+^ (94%), HPV33^+^ (100%) and 35^+^ (100%), cancer, whereas a lower detection rate was observed among the two patients with HPV18^**+**^ (50%) cancer, which is in line with data in HPV^+^ cervical cancer (unpublished data). Of note, the HPV18^**+**^ TSCC patient with a negative cfHPV-DNA in plasma at diagnosis had a T1N0M0 tumor.

Moreover, variations in cfHPV-DNA positivity in the diagnostic samples were observed across HNC subtypes. Patients with HPV^+^ OPSCC or HPV^+^ HNCUP were more likely to be cfHPV-DNA-positive in their diagnostic sample as compared to those with other HPV^+^ HNC subtypes.

However, due to the limited sample size, further studies are needed to validate these findings.

On the other hand, in one patient with an HPV35^**+**^ nasopharyngeal cancer, the regression of disease could be followed, while in the patient with an HPV16^**+**^ lacrimal gland tumor, regarded as cfHPV-DNA-negative in plasma at diagnosis, it was possible to observe increasing values of cfHPV-DNA at later time points. A third patient with an HPV16^**+**^ nasopharyngeal squamous cell carcinoma lacked cfHPV-DNA in plasma during the whole observation period, but this tumor was also a T1N0M0, which could explain the lack of cfHPV-DNA.

In contrast to the small number of patients with HPV^+^ non-OPSCC cancer, in the HPV^+^ HNCUP group the proportion cfHPV-DNA-positive diagnostic samples was high, suggesting that cfHPV-DNA detection could serve as a valuable tool for diagnosis and monitoring in this subgroup^51^.

It is also noteworthy that the only two organ transplanted patients included during the observation period did not fare well. How organ transplant patients should be treated under the present circumstances is a topic for further studies. It may or may not be worthwhile to examine the possibility of regularly screening organ transplant patients by cfHPV-DNA monitoring e.g. once per year, in order to facilitate earlier detection of an HPV^+^OPSCC or HPV^+^ HNC or other HPV^+^ cancer. However, this is not a straightforward possibility either, since there are still extensive knowledge gaps in the field^52^. In addition we do not presently know whether organ transplant patients may disclose a different cfHPV-DNA profile compared to patients who are not immunocompromised.

This study has limitations. So far, patients have only been followed-up for just over one year, and clearly this report it cannot serve as a validation for clinical use, where many aspects still need to be addressed as presented by Kuh et al.^52^. Moreover, clinical recurrences in patients diagnosed with primary tumors were not encountered during the duration of this study. This was however not unexpected since the follow-up time was limited and relapses generally come later. Nevertheless, during this short period we have been able to monitor therapy resistance in one patient at least, while we have observed an increase in cfHPV-DNA levels in another patient. Separately, in the patient with a distant recurrence at recruitment, we could demonstrate that the initially high cfHPV-DNA levels decreased as the patient responded to therapy.

Another limitation was that there were too few HPV^+^ non-OPSCC or patients with HPV18^**+**^ cancer to draw conclusions on the utility of cfHPV-DNA analysis in these subgroups. Therefore, additional studies here are warranted to resolve these issues.

A further limitation was that the initial tumor biopsies were not assessed for viral integration, episomal copies/viral load or tumor necrosis was also a limitation, since HPV copy numbers in the tumor may affect cfHPV detectability as shown previously^46^. Likewise, tumor necrosis may also affect what is found initially or may occur early on upon treatment, e.g. in “early peakers”^46,47^.

Finally, it could be of particular interest to reflect on the threshold level for what constitutes a positive cfHPV-DNA value. In two cases, we observed a discrepancy between the cfHPV-DNA finding and the clinical picture. For example, in one patient, the cfHPV-DNA level was 0.7 copies/ml (considered negative) at 3 months, yet this patient had an HPV^+^ lymph node exhibiting residual metabolic activity on PET-CT imaging. Perhaps, this sample could have been reported as inconclusive and a new sample (preferably with a larger volume of plasma) could be taken within a month in order to confirm or exclude persistent tumour burden. The latter could be of special value, in light of further details, supporting the use of cfHPV-DNA as compared to PET-CT for detecting residual metabolic activity as reported by Tanaka et al.^53^.

In conclusion, cfHPV-DNA was detectable in the plasma of most patients with HPV^+^ positive OPSCC and HNCUP at diagnosis, using ddPCR, while this was not consistently the case for patients with HPV^+^ non-OPSCC HNC. Notably, cfHPV-DNA became undetectable in nearly one-third of patients within 3–4 weeks of initiating RT and in most patients by 3 months following treatment completion. Nevertheless, clearance patterns varied by tumor subtype and HPV genotype. We conclude that cfHPV-DNA is a potential biomarker of treatment response. However, larger studies with a broader spectrum of HPV^+^ HNC subtypes, diverse HPV genotypes and extended follow-up periods are warranted to determine its prognostic significance more robustly.

## Data Availability

The data sets (anonymized individual-level data) generated and/or analysed during this current study are available from the corresponding author upon reasonable request.
